# Radiological Outcomes of PEEK Versus Rigid Rod Stabilization in Lumbar Spinal Stenosis Surgery: The Role of Preoperative and Postoperative Findings in Adjacent Segment Disease

**DOI:** 10.3390/diagnostics16111625

**Published:** 2026-05-26

**Authors:** Merih Can Yilmaz, Ozgur Ozaydin, Keramettin Aydin

**Affiliations:** 1 Department of Neurosurgery, VM Medical Park Hospital, 55200 Samsun, Turkey; keramettinns@gmail.com; 2 Department of Economics, Ondokuz Mayıs University, 55270 Samsun, Turkey; ozaydin@omu.edu.tr

**Keywords:** PEEK rod, rigid rod, adjacent segment disease

## Abstract

**Background/Objectives:** Lumbar spondylosis is a degenerative disorder that may require decompression and stabilization surgery. Rigid titanium rods provide strong fixation, whereas polyetheretherketone (PEEK) rods have been proposed to offer a more flexible load distribution profile. This study compared the radiological outcomes after PEEK versus rigid rod stabilization and evaluated whether the preoperative degenerative findings contributed independently to the postoperative adjacent segment radiological status. **Methods:** A retrospective cohort of 106 patients undergoing lumbar decompression and posterior stabilization (2020–2025) was analyzed. Rod allocation followed routine clinical practice rather than randomization. Radiological parameters (foraminal area, canal diameter, disc height, and facet volume) were measured preoperatively and at one year postoperatively. Baseline-adjusted ANCOVA models with HC3 robust inference compared PEEK and rigid rods across the two-, three-, and four-segment constructs. Additional models assessed the independent effects of preoperative facet effusion and Modic changes. **Results:** PEEK rods were associated with the statistically reliable preservation of spinal canal diameter, foraminal area, disc height (particularly in the three- and four-segment constructs), and reduced facet joint volume increase compared with rigid rods after multiple-comparison correction. The findings for the two-segment constructs were less consistent and partly influence-sensitive. Preoperative facet effusion and Modic changes showed no statistically reliable independent association with postoperative radiological outcomes after adjustment. **Conclusions:** PEEK rod systems were associated with favorable baseline-adjusted radiological preservation patterns, especially in long-segment stabilization. These findings should be interpreted as radiological associations rather than proof of clinical superiority or causal reduction in adjacent segment disease, because rod allocation was nonrandom and clinical, and fusion-related outcomes were not assessed.

## 1. Introduction

Lumbar spondylosis is a chronic process characterized by age-related degenerative changes in the lumbar spine, including intervertebral disc degeneration, facet joint hypertrophy, and ligamentum flavum thickening [1]. These structural changes can lead to narrowing of the spinal canal and neural foramina over time, resulting in clinical conditions such as lower back pain, radiculopathy, and neurogenic claudication. As the degenerative process progresses, segmental instability may develop, exacerbating the severity of symptoms.

While conservative treatment methods (analgesic therapy, physical therapy, and injections) constitute the first-line approach for most patients, surgical treatment becomes necessary in the presence of progressive neurological deficits, severe spinal stenosis, or significant instability. Laminectomy, performed in this context, provides decompression of the neural structures through removal of posterior elements. However, decompression alone may not be sufficient in cases of concomitant instability. Therefore, posterior instrumentation using pedicle screws and rod systems is frequently added to surgery to ensure segmental stability and to prevent deformity [2,3,4,5,6].

The combination of laminectomy and instrumentation is considered to be an effective surgical approach in terms of reducing pain, achieving neurological recovery, and improving quality of life when applied in appropriate indications [7,8].

While the traditionally-used titanium rigid rod systems provide strong stabilization, they may increase the stress transmitted to neighboring segments and create a more biomechanically rigid structure [9].

In recent years, more flexible rod systems made of polyetheretherketone (PEEK) material have been developed to provide a more physiological load distribution profile. PEEK rods have a lower elastic modulus than titanium rigid rod systems, and biomechanical and clinical reports have suggested that this difference may reduce stress transfer to adjacent segments [4,5,6,7]. However, whether these theoretical biomechanical advantages translate into measurable postoperative radiological preservation remains uncertain.

The aim of this study is to compare the retrospective radiological outcomes of PEEK versus rigid rods in patients who had undergone surgical treatment for lumbar spondylosis and to evaluate whether preoperative facet effusion and Modic changes independently contributed to the postoperative adjacent segment radiological status. We hypothesized that, after adjustment for baseline radiological status and patient covariates, PEEK rods would be associated with more favorable postoperative radiological preservation than rigid rods, particularly in longer constructs, and that preoperative degenerative findings would not fully account for the observed rod material differences.

## 2. Materials and Methods

### 2.1. Study Design and Patient Population

This retrospective, single-center study included patients with lumbar spondylosis/lumbar spinal stenosis who had undergone lumbar decompression and posterior stabilization between 2020 and 2025 and had complete preoperative and postoperative radiological imaging. Postoperative imaging was evaluated at the end of the first postoperative year (+/−2 months). The analysis compared the adjacent segment foraminal area, spinal canal anteroposterior diameter, lumbar disc sagittal plane height, and mean facet volume.

Patients with oncological lumbar spine disease (e.g., metastasis), traumatic lumbar spine fracture, or anterior/interbody fusion material, including transforaminal lumbar interbody fusion (TLIF) or posterior lumbar interbody fusion (PLIF), were excluded. No included patient underwent interbody fusion. Preoperative facet effusion and Modic changes at the adjacent segment were evaluated as prespecified radiological degenerative markers.

### 2.2. Surgical Technique and Rod Selection

The rod material was selected in routine clinical practice and was not randomized. Selection was influenced by economic considerations, patient age, adjacent segment degeneration neighboring the planned construct, the overall biomechanical plan, and spondylolisthesis severity. In particular, rigid rods were generally preferred in patients with grade 2 or higher spondylolisthesis. This nonrandom allocation was considered a potential source of confounding by indication.

All operations were performed by the same two-surgeon team working together during the procedure. The standard surgical approach consisted of bilateral hemilaminectomy and posterior segmental instrumentation/stabilization. In rigid rod cases, autogenous graft was used adjacent to the screws. Postoperative rehabilitation followed the same standardized pathway for all patients.

### 2.3. Radiological Assessment and Outcomes

Radiological measurements were performed on preoperative magnetic resonance imaging (MRI) or computed tomography (CT), and postoperative MRI or CT images used the hospital software program Synapse v7.1.000. The bilateral foraminal areas and facet volumes at the adjacent segment were averaged for analysis. Measurements were performed by the corresponding author and were not blinded because the rod material was visible on postoperative MRI/CT images, making complete blinding impractical. Interobserver and intraobserver reliability were not assessed.

This study was limited to radiological outcomes. Clinical outcome measures, including visual analog scale scores, Oswestry Disability Index, Japanese Orthopaedic Association scores, reoperation, complications, and functional recovery, were not available for analysis. Fusion status, pseudarthrosis, and screw loosening were also not assessed; the prespecified outcome focus was the adjacent segment radiological status.

### 2.4. Ethics

Ethical approval was obtained from the Institutional Ethics Committee of Ondokuz Mayıs University Faculty of Medicine Hospital for the retrospective analysis of existing clinical and radiological records (application number 2026/36, approval date 9 February 2026). Radiological measurements for the present study were completed after ethics approval. In institutional routine practice, surgically treated patients provide surgical consent as well as consent for the use of clinical and radiological data in academic research.

### 2.5. Statistical Methodology

#### 2.5.1. Analysis Cohort and Data Structure

The analysis used an analysis-ready workbook containing 106 patients in the patient-level wide dataset and 212 observations in the long-format repeated-measures dataset. The wide dataset contained one row per patient and was used for all primary baseline-adjusted postoperative analyses. The long dataset contained exactly two observations per patient, preoperative and postoperative, and was used only for supportive repeated-measures mixed models. Construct groups were coded as 2P, 2R, 3P, 3R, 4P, and 4R, where the numeric component denotes the construct length and P/R denotes PEEK or rigid rod material.

The radiological endpoints were foraminal measurement, spinal canal measurement, disc height measurement, and facet joint volume. For foraminal, canal, and disc measurements, higher postoperative values were interpreted as more favorable preservation. For the facet joint volume, a postoperative increase was interpreted as radiological deterioration; therefore, negative PEEK-minus-rigid estimates favor PEEK for facet volume, whereas positive PEEK-minus-rigid estimates favor PEEK for foraminal, canal, and disc endpoints.

No imputation was performed. The endpoint-specific primary models used a complete-case analysis for the variables required by that endpoint. The analysis population files documented zero missing-data exclusions for the primary model variables.

#### 2.5.2. Primary Baseline-Adjusted Models

The primary inferential backbone was an HC3-robust baseline-adjusted postoperative ANCOVA framework. For rod material comparisons within construct length, each postoperative endpoint was modeled as a function of its corresponding preoperative value, rod material, construct length, the rod-material-by-construct-length interaction, age, and sex. Segment-specific PEEK-minus-rigid contrasts were then estimated within the two-, three-, and four-segment strata.

To evaluate whether the preoperative degenerative findings independently contributed to the postoperative radiological status, a second baseline-adjusted additive model was fitted for each endpoint. This model included the corresponding preoperative endpoint value, preoperative facet effusion, preoperative Modic change, rod material, construct length, the rod-material-by-construct-length term, age, and sex. Preoperative facet effusion and Modic change were retained as separate main effect predictors in the primary additive model.

All *p*-values were two-sided, the nominal alpha level was 0.05, and all confidence intervals were 95% confidence intervals. Reported confidence intervals are HC3 robust Wald confidence intervals unless otherwise stated. Multiplicity adjustment was applied to *p*-values, not to confidence intervals. The primary multiplicity correction was the global Holm correction within each prespecified primary family: 12 contrasts for the rod-material-by-construct-length comparison family and 8 contrasts for the preoperative degenerative marker family. Endpoint-specific Holm-adjusted *p*-values were retained only as supplemental transparency and were not used as the primary inferential column.

#### 2.5.3. Supportive, Sensitivity, and Exploratory Analyses

Supportive mixed models were fitted in the long format dataset using random intercepts for patients. These models assessed whether the pre-to-postoperative change patterns were directionally consistent with the primary baseline-adjusted postoperative contrasts. Timepoint-by-covariate terms were used so that time-invariant covariates could contribute to the trajectory adjustment. Mixed-model estimates were treated as supportive consistency checks and did not supersede the primary ANCOVA estimands.

Paired *t*-tests and Wilcoxon signed-rank tests summarized the within-group pre-to-postoperative changes and were considered descriptive only (Supplementary Table S13). They were not used as between-group evidence and were not treated as fallback analyses.

Sensitivity analyses were prespecified as interpretation aids and did not replace the primary estimates. Cook’s distance influence-trimmed refits assessed robustness to influential observations. Baseline slope heterogeneity was assessed across endpoints; where detected, conditional contrasts were used to describe the baseline-dependent behavior. Diagnostic flags, including heteroskedasticity, residual non-normality, and influential observations, were treated as markers requiring cautious interpretation rather than as automatic invalidation of the primary models.

For the preoperative degenerative marker analysis, the rod-material-by-Modic interaction inference was not claim-bearing because Modic-positive patients were sparse and a zero cell was present in one rod group stratum. The preoperative Modic main effect nevertheless remained part of the primary additive model. The preoperative facet-effusion-by-rod-material interaction was considered exploratory only. The composite any-preoperative-degenerative-finding variable was used only as a sparsity sensitivity analysis and did not replace the separate marker primary model. Reproducibility and output-validation checks, including data-sheet equivalence, expected-output validation, and table-shell compliance for the rod/construct and degenerative-marker analyses, are summarized in Supplementary Table S14.

## 3. Results

### 3.1. Cohort Characteristics and Baseline Balance

The study cohort included 106 patients, 11 in 2P, 16 in 2R, 18 in 3P, 32 in 3R, 12 in 4P, and 17 in 4R. The baseline characteristics and preoperative radiological measurements are summarized in Table 1. Baseline imbalance was present across several strata, including sex distribution in the two-segment and four-segment comparisons, age in the three-segment comparison, and preoperative canal measurement in the four-segment comparison; these imbalances supported the use of baseline-adjusted modeling rather than unadjusted postoperative or change-score comparisons (Supplementary Table S1; Supplementary Figures S1 and S2).

### 3.2. Adjusted Postoperative Rod Material Comparisons

**Figure 1:**
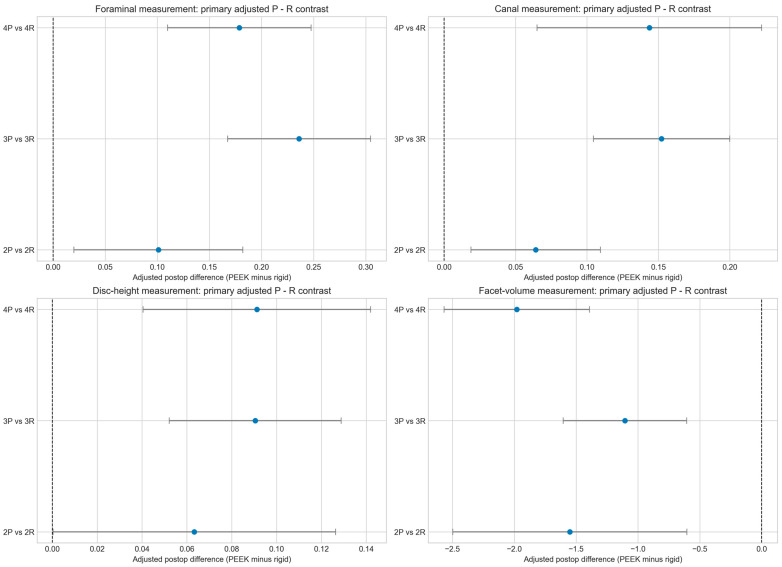
Primary adjusted PEEK-minus-rigid contrasts by endpoint and construct length.

After baseline adjustment and global Holm correction, the PEEK constructs showed statistically reliable preservation of spinal canal measurements across the two-, three-, and four-segment strata. The adjusted PEEK-minus-rigid differences were 0.064 in the two-segment constructs, 0.152 in the three-segment constructs, and 0.144 in the four-segment constructs (all global Holm-adjusted *p*-values < 0.05; Table 2; Figure 1). Adjusted postoperative marginal means by construct group are provided in Supplementary Figure S3.

The disc height findings were most robust in longer constructs. The adjusted differences favored PEEK in the three- and four-segment constructs and remained statistically reliable after global Holm correction. The two-segment disc height contrast reached the global Holm threshold but was classified as borderline and influence-sensitive; after influence trimming, the corresponding contrast was smaller and no longer statistically reliable. This finding should therefore be interpreted as suggestive rather than robust.

Facet volume contrasts favored PEEK in all construct length strata, with negative PEEK-minus-rigid estimates indicating less postoperative facet volume increase relative to the rigid constructs. The three- and four-segment facet findings were statistically reliable and not classified as influence-sensitive. The two-segment facet contrast was statistically reliable after global Holm correction but influence-sensitive, warranting cautious interpretation (Supplementary Table S7).

Foraminal measurements favored PEEK in all construct length strata after global Holm correction. However, a baseline slope heterogeneity test was significant for the foramen endpoint; therefore, the single primary contrast should be interpreted together with the conditional sensitivity estimates. Conditional contrasts were small and not statistically reliable at the 25th percentile of the baseline foramen in the two-segment stratum but favored PEEK at the median and 75th percentile, and consistently favored PEEK across the three- and four-segment strata (Supplementary Table S6; Supplementary Figure S4). Supportive repeated-measures mixed-model analyses showed directionally consistent PEEK-minus-rigid difference-in-change patterns for rod-material comparisons and are provided in Supplementary Table S9 and Supplementary Figure S5.

### 3.3. Independent Associations of Preoperative Degenerative Findings

Preoperative facet effusion was present in 30 patients (28.3%), preoperative Modic change was present in 12 patients (11.3%), and at least one of these findings was present in 34 patients (32.1%). Modic-positive counts were sparse, including zero Modic-positive patients in the 3P group; therefore, the rod-material-by-Modic interaction inference was not treated as claim-bearing (Supplementary Tables S2–S4; Supplementary Figures S6 and S7).

**Figure 2:**
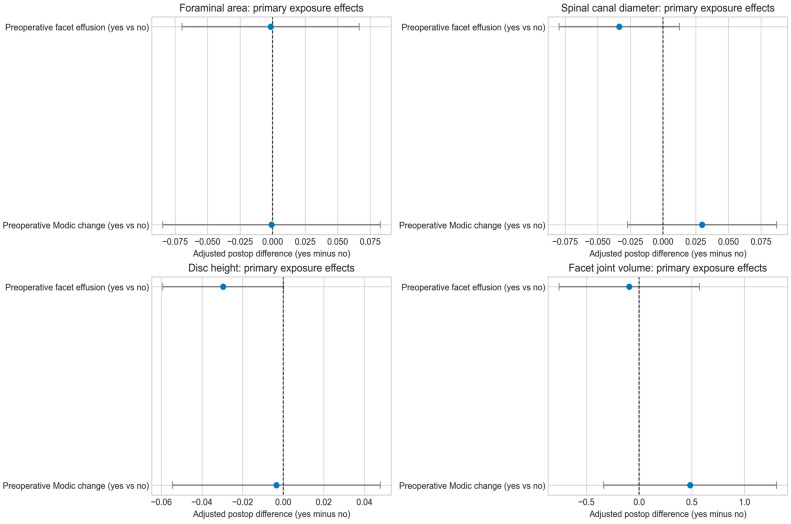
Primary adjusted associations of preoperative degenerative markers with postoperative radiological endpoints.

In the primary additive models, no statistically reliable independent association was detected between either preoperative facet effusion or preoperative Modic change and any postoperative radiological endpoint after global Holm correction (Table 3; Figure 2). The closest nominal signal was for disc height with preoperative facet effusion (beta, −0.030; 95% CI, −0.059 to 0.000; nominal *p* = 0.051; global Holm *p* = 0.407), but this did not meet the prespecified primary inferential criterion and should not be presented as confirmatory.

Supportive and exploratory analyses were directionally informative but did not alter the primary interpretation. The supportive mixed model showed a nominal association between preoperative facet effusion and disc-height change, and the exploratory facet-effusion-by-rod-material interaction was nominally positive for disc height. Both findings remained outside the claim-bearing primary analysis set. The composite any-preoperative-degenerative-finding sensitivity analysis similarly did not identify a statistically reliable association after Holm correction (Supplementary Tables S8–S12; Supplementary Figures S8 and S9).

### 3.4. Model Diagnostics and Sensitivity Interpretation

Heteroskedasticity was detected for several primary models, supporting the prespecified use of HC3 robust inference. Residual non-normality was most evident for disc and facet outcomes. Influential observations were detected across endpoints, but influence-trimmed analyses generally supported the longer-construct findings (Supplementary Table S7). The main exceptions were the two-segment disc contrast and the two-segment facet contrast, which were classified as influence-sensitive and should be described cautiously. These diagnostic findings do not invalidate the primary ANCOVA estimates but identify endpoints where sensitivity results should temper interpretation (Supplementary Table S5; Supplementary Figures S10–S17).

## 4. Discussion

This retrospective radiological study found that PEEK rods were associated with more favorable baseline-adjusted postoperative radiological preservation than rigid rods across several endpoint and construct length comparisons. The most consistent findings were observed in the longer constructs, particularly for canal diameter, disc height, and facet volume change. By contrast, some of the two-segment findings were classified as borderline or influence-sensitive, and should be interpreted cautiously. Preoperative facet effusion and Modic changes were not statistically reliable independent predictors of postoperative radiological status after adjustment.

The rationale for using PEEK rods is based on their lower elastic modulus relative to titanium and the theoretical possibility of reducing stress transfer to adjacent segments [9,10,11,12]. Rigid rods provide strong mechanical stabilization, whereas semirigid constructs may alter load distribution. The present study did not directly measure spinal motion, dynamic stability, or biomechanical load sharing; therefore, the findings should be interpreted as radiological associations compatible with this rationale, not as direct evidence that PEEK rods preserved physiological motion.

Previous clinical and biomechanical studies comparing PEEK and titanium constructs in lumbar surgery have reported the potential advantages of PEEK rods for postoperative function, adjacent segment behavior, and construct biomechanics [13,14,15]. More recent finite element studies have further provided biomechanical plausibility that semirigid PEEK constructs may influence stiffness, load sharing, and adjacent-level mechanical responses, while smaller clinical and radiological series have reported favorable postoperative or adjacent segment observations with PEEK-based constructs [16,17,18,19]. However, this favorable interpretation is not uniform across the literature; registry-based studies and systematic reviews have also reported mixed findings, including studies that did not demonstrate consistent material superiority or a clear reduction in adjacent segment disease with PEEK rods [20,21,22]. Our results add to this literature by using baseline-adjusted, construct length-specific radiological comparisons and by separating primary confirmatory inference from supportive, exploratory, and sensitivity analyses. Nevertheless, the present results remain observational and radiological in scope.

Rod selection in this cohort reflected routine clinical practice rather than random assignment. Economic considerations, patient age, the degree of degeneration near the planned construct, the biomechanical surgical plan, and spondylolisthesis severity influenced the choice of rod material, with rigid rods generally preferred in grade 2 or higher spondylolisthesis. Although the primary models were adjusted for baseline radiological status, age, sex, and construct length, residual confounding by indication cannot be excluded.

The absence of a statistically reliable independent association between preoperative facet effusion or Modic change and postoperative radiological endpoints should also be interpreted conservatively. The Modic-positive subgroup was small and included a zero-cell stratum, which prevented a claim-bearing interaction inference. These findings therefore do not prove that preoperative degenerative markers are clinically irrelevant; rather, they indicate that no statistically reliable independent radiological association was detected within this dataset and model framework.

### Limitations

This study has several limitations. First, the retrospective single-center design and nonrandom rod allocation introduce selection bias and is confounding by indication. Second, subgroup sample sizes were modest, and sparse Modic-positive cells limited interaction analyses. Third, this was a radiological-only study: patient-reported outcomes, pain scores, disability scores, reoperation, complications, functional recovery, fusion status, pseudarthrosis, and screw loosening were not assessed. Radiological preservation therefore should not be interpreted as proof of clinical benefit. Fourth, radiological measurements were performed by a single non-blinded observer, the corresponding author. Blinding was impractical because the rod material was visible on postoperative MRI/CT images, but the lack of blinding and the absence of an interobserver or intraobserver reliability assessment may introduce measurement-related bias. Fifth, dynamic imaging and direct biomechanical assessments were not performed, so this study cannot establish whether PEEK rods preserved physiological motion. Finally, influence-sensitive findings, especially in the two-segment constructs, should be regarded as hypothesis-generating rather than definitive.

## 5. Conclusions

In this retrospective radiological cohort, PEEK rods were associated with more favorable baseline-adjusted postoperative radiological estimates for foraminal area, canal diameter, disc height, and facet volume, most consistently in the three- and four-segment constructs. Findings in the two-segment constructs were less robust and should be interpreted cautiously. Given the less robust and influence-sensitive findings in the two-segment constructs, the radiological data from this study do not support a blanket interpretation that PEEK rods should be preferred across all construct lengths; material selection in the two-segment constructs should remain individualized and guided by clinical, biomechanical, and resource-related considerations. No statistically reliable independent association was detected between preoperative facet effusion or Modic change and postoperative radiological endpoints after adjustment. The rod material-related radiological patterns were not fully attenuated after inclusion of these preoperative degenerative markers in the additive models. These findings support further prospective research on rod material-related radiological outcomes, but they do not establish clinical superiority, equivalence, non-inferiority, or a causal reduction in adjacent segment disease.

## Figures and Tables

**Table 1:** Baseline characteristics and preoperative radiological measurements by construct group.

Group	*n*	Age, Mean (SD)	Female	Facet Effusion	Modic	Foramen Preop	Canal Preop	Disc Preop	Facet Preop
2P	11	57.545 (8.079)	63.6%	18.2%	9.1%	0.565 (0.142)	1.025 (0.180)	0.820 (0.316)	6.333 (1.586)
2R	16	50.625 (18.257)	12.5%	18.8%	18.8%	0.682 (0.219)	1.107 (0.188)	0.964 (0.190)	6.188 (2.091)
3P	18	44.500 (11.068)	44.4%	22.2%	0.0%	0.867 (0.223)	1.203 (0.156)	0.947 (0.155)	5.725 (1.493)
3R	32	55.750 (15.656)	65.6%	37.5%	9.4%	0.790 (0.226)	1.294 (0.288)	0.958 (0.244)	5.193 (1.491)
4P	12	58.417 (9.986)	91.7%	33.3%	8.3%	0.632 (0.158)	1.100 (0.118)	0.886 (0.218)	5.192 (1.790)
4R	17	61.176 (10.984)	64.7%	29.4%	23.5%	0.782 (0.292)	1.299 (0.238)	0.822 (0.215)	5.298 (1.531)

Values are mean (SD) or percentage. P denotes PEEK rod; R denotes rigid rod.

**Table 2:** Primary HC3-robust baseline-adjusted postoperative PEEK-minus-rigid contrasts by construct length.

Endpoint	Comparison	n P	n R	Preop P	Preop R	Adj Diff	95% CI	*p*	Global Holm *p*	Primary Decision
canal	2P vs. 2R	11	16	1.025 (0.180)	1.107 (0.188)	0.064	0.0188 to 0.1095	0.006	0.017	Reliable after global Holm
canal	3P vs. 3R	18	32	1.203 (0.156)	1.294 (0.288)	0.152	0.1045 to 0.1999	<0.001	<0.001	Reliable after global Holm
canal	4P vs. 4R	12	17	1.100 (0.118)	1.299 (0.238)	0.144	0.0650 to 0.2223	<0.001	0.002	Reliable after global Holm
disc	2P vs. 2R	11	16	0.820 (0.316)	0.964 (0.190)	0.063	0.0003 to 0.1263	0.049	0.049	Borderline; influence-sensitive
disc	3P vs. 3R	18	32	0.947 (0.155)	0.958 (0.244)	0.090	0.0521 to 0.1288	<0.001	<0.001	Reliable after global Holm
disc	4P vs. 4R	12	17	0.886 (0.218)	0.822 (0.215)	0.091	0.0405 to 0.1420	<0.001	0.002	Reliable after global Holm
facet	2P vs. 2R	11	16	6.333 (1.586)	6.188 (2.091)	−1.551	−2.4976 to −0.6054	0.001	0.005	Borderline; influence-sensitive
facet	3P vs. 3R	18	32	5.725 (1.493)	5.193 (1.491)	−1.105	−1.6045 to −0.6061	<0.001	<0.001	Reliable after global Holm
facet	4P vs. 4R	12	17	5.192 (1.790)	5.298 (1.531)	−1.979	−2.5674 to −1.3914	<0.001	<0.001	Reliable after global Holm
foramen	2P vs. 2R	11	16	0.565 (0.142)	0.682 (0.219)	0.101	0.0201 to 0.1821	0.014	0.029	Reliable after global Holm
foramen	3P vs. 3R	18	32	0.867 (0.223)	0.790 (0.226)	0.236	0.1674 to 0.3047	<0.001	<0.001	Reliable after global Holm
foramen	4P vs. 4R	12	17	0.632 (0.158)	0.782 (0.292)	0.179	0.1099 to 0.2475	<0.001	<0.001	Reliable after global Holm

Primary inference is based on the global Holm-adjusted *p*-value. Positive estimates favor PEEK for foramen, canal, and disc endpoints; negative estimates favor PEEK for facet volume.

**Table 3:** Primary HC3-robust additive associations of preoperative facet effusion and Modic change with postoperative radiological endpoints.

Endpoint	Predictor	Beta	95% CI	*p*	Global Holm *p*	Primary Decision	Sparse
canal	facet effusion (yes vs. no)	−0.034	−0.0796 to 0.0125	0.153	1.000	Not reliable after global Holm	yes
canal	Modic change (yes vs. no)	0.030	−0.0271 to 0.0870	0.304	1.000	Not reliable after global Holm	yes
disc	facet effusion (yes vs. no)	−0.030	−0.0593 to 0.0001	0.051	0.407	Not reliable after global Holm	yes
disc	Modic change (yes vs. no)	−0.003	−0.0545 to 0.0479	0.898	1.000	Not reliable after global Holm	yes
facet	facet effusion (yes vs. no)	−0.093	−0.7583 to 0.5722	0.784	1.000	Not reliable after global Holm	yes
facet	Modic change (yes vs. no)	0.485	−0.3359 to 1.3053	0.247	1.000	Not reliable after global Holm	yes
foramen	facet effusion (yes vs. no)	−0.002	−0.0696 to 0.0666	0.966	1.000	Not reliable after global Holm	yes
foramen	Modic change (yes vs. no)	−0.001	−0.0844 to 0.0828	0.985	1.000	Not reliable after global Holm	yes

Primary inference is based on global Holm-adjusted *p*-values. The Modic main effect remains part of the primary model; rod-material-by-Modic interaction inference is not claim-bearing because of sparse and zero cells.

## Data Availability

The original contributions presented in this study are included in the article/supplementary material. Further inquiries can be directed to the corresponding author.

## Supplementary Materials

The following supporting information can be downloaded at: https://www.mdpi.com/article/10.3390/diagnostics16111625/s1. Supplementary Table S1. Baseline standardized mean differences for PEEK versus rigid comparisons within construct length. Supplementary Table S2. Overall prevalence of preoperative degenerative markers. Supplementary Table S3. Sparsity and interaction feasibility profile for preoperative degenerative markers. Supplementary Table S4. Interaction feasibility decisions. Supplementary Table S5. Primary model diagnostic flags. Supplementary Table S6. Conditional foramen contrasts across prespecified baseline levels. Supplementary Table S7. Influence-trimmed sensitivity for PEEK-minus-rigid contrasts. Supplementary Table S8. Influence-trimmed sensitivity for preoperative degenerative-marker associations. Supplementary Table S9. Supportive mixed-model difference-in-change contrasts for rod-material comparisons. Supplementary Table S10. Supportive mixed-model difference-in-change contrasts for preoperative degenerative markers. Supplementary Table S11. Exploratory facet-effusion-by-rod-material interaction terms. Supplementary Table S12. Composite any-preoperative-degenerative-finding sensitivity analysis. Supplementary Table S13. Descriptive within-group paired pre-to-postoperative tests. Supplementary Table S14. Reproducibility and output validation checks. Supplementary Figure S1. Crude preoperative-to-postoperative mean profiles by construct group. Supplementary Figure S2. Change-score distributions by construct group. Supplementary Figure S3. Adjusted postoperative marginal means by construct group. Supplementary Figure S4. Baseline-dependent foramen sensitivity estimates. Supplementary Figure S5. Supportive mixed-model PEEK-minus-rigid difference-in-change contrasts. Supplementary Figure S6. Preoperative degenerative-marker prevalence by construct group. Supplementary Figure S7. Overlap of preoperative facet effusion and Modic change. Supplementary Figure S8. Exploratory adjusted margins for facet effusion by rod material. Supplementary Figure S9. Foramen exposure-effect sensitivity comparison. Supplementary Figure S10. Diagnostic plots for the foramen rod-material model. Supplementary Figure S11. Diagnostic plots for the canal rod-material model. Supplementary Figure S12. Diagnostic plots for the disc rod-material model. Supplementary Figure S13. Diagnostic plots for the facet rod-material model. Supplementary Figure S14. Diagnostic plots for the foramen degenerative-marker model. Supplementary Figure S15. Diagnostic plots for the canal degenerative-marker model. Supplementary Figure S16. Diagnostic plots for the disc degenerative-marker model. Supplementary Figure S17. Diagnostic plots for the facet degenerative-marker model.

## Abbreviations

The following abbreviations are used in this manuscript:

PEEK	Polyetheretherketone
MRI	Magnetic Resonance Imaging
CT	Computed Tomography
TLIF	Transforaminal Lumbar Interbody Fusion
PLIF	Posterior Lumbar Interbody Fusion
ANCOVA	Analysis of Covariance
HC3	Heteroskedasticity-Consistent
