# Supplementary material for: Radiological Outcomes of PEEK Versus Rigid Rod Stabilization in Lumbar Spinal Stenosis Surgery: The Role of Preoperative and Postoperative Findings in Adjacent Segment Disease
Source: Diagnostics (Basel). 2026 May 26;16(11):1625. doi: 10.3390/diagnostics16111625 (PMC13256108; doi:10.3390/diagnostics16111625)

Supplementary Materials

The supplementary materials below document the baseline balance, model diagnostics, sensitivity analyses, supportive mixed models, sparsity checks, and reproducibility controls. These materials are subordinate to the primary HC3-robust baseline-adjusted analyses and are intended to support a transparent review.

Scope note: These supplementary materials support a radiological-only analysis. Clinical outcomes, fusion status, pseudarthrosis, screw loosening, and interobserver/intraobserver reliability metrics were not available or assessed. Radiological measurements were performed by the corresponding author and were not blinded because the rod material was visible on postoperative MRI/CT images.

Supplementary Table S1. Baseline standardized mean differences for PEEK versus rigid comparisons within construct length.

segments_n	age_smd_P_vs_R	sex_F_smd_P_vs_R	preop_facet_eff_yes_smd_P_vs_R	preop_modic_yes_smd_P_vs_R	preop_foramen_smd_P_vs_R	preop_canal_smd_P_vs_R	preop_disc_smd_P_vs_R	preop_facet_smd_P_vs_R
2.0	0.4602409022528014	1.053153945497988	-0.014643077771249	-0.2790355663875498	-0.6096207626845528	-0.4456000286998233	-0.581742409425155	0.0758912158846346
3.0	-0.792180680049381	-0.4257751567712715	-0.3338318476565498	-0.4435327625727437	0.3405166060407828	-0.3629422304823379	-0.0502946808100791	0.3565759242217186
4.0	-0.2606310300264899	0.6528333621091074	0.0845154254728516	-0.4152292616883817	-0.6076356822611735	-1.003842315465702	0.2932528976592432	-0.064560134114729

Supplementary Table S2. Overall prevalence of preoperative degenerative markers.

Variable	Yes n	No n	Yes %
preop_facet_eff	30	76	28.3%
preop_modic	12	94	11.3%
any_preop_deg	34	72	32.1%

Supplementary Table S3. Sparsity and interaction feasibility profile for preoperative degenerative markers.

Variable	Yes n	No n	Min cell	Zero cells	Zero-cell group	Interaction feasibility	Claim scope
preop_facet_eff	30	76	2	0		limited; use caution for interaction inference	exploratory only for interaction
preop_modic	12	94	0	1	3P:preop_modic=yes	not claim-bearing for rod_material x preop_modic interaction	not for primary inference for interaction; main effect remains primary
any_preop_deg	34	72	2	0		limited; use caution for interaction inference	exploratory only for interaction

Supplementary Table S4. Interaction feasibility decisions.

candidate_interaction	minimum_cell_n	zero_cells_present	zero_cell_groups	status	reason	claim_scope
rod_material x preop_facet_eff	2	False		exploratory only	Limited one-at-a-time effect-modification assessment; not claim-bearing.	exploratory only
rod_material x preop_modic	0	True	3P:preop_modic=yes	not part of claim-bearing analysis set	Sparse and zero cells make the interaction estimate unstable and not interpretable for formal inference.	not for primary inference

Supplementary Table S5. Primary model diagnostic flags.

Analysis	Endpoint	n	BP p	Shapiro p	Max Cook D	Cook >4/n	Hetero	Non-normal	Influence
Rod/construct comparison	canal	106	<0.001	0.064	0.091	8	yes	no	yes
Rod/construct comparison	disc	106	0.011	0.005	0.153	6	yes	yes	yes
Rod/construct comparison	facet	106	0.065	<0.001	0.194	7	no	yes	yes
Rod/construct comparison	foramen	106	0.005	0.256	0.106	8	yes	no	yes
Preoperative marker model	canal	106	<0.001	0.107	0.098	7	yes	no	yes
Preoperative marker model	disc	106	0.034	<0.001	0.135	7	yes	yes	yes
Preoperative marker model	facet	106	0.114	<0.001	0.295	7	no	yes	yes
Preoperative marker model	foramen	106	0.005	0.266	0.154	8	yes	no	yes

BP denotes Breusch–Pagan test. Flags are interpretation aids and do not automatically invalidate the primary estimates.

Supplementary Table S6. Conditional foramen contrasts across prespecified baseline levels.

Segments	Baseline percentile	Baseline value	Adj P-R	p	95% CI
2	25th	0.475	0.039	0.263	-0.030 to 0.108
2	50th	0.64	0.113	0.008	0.030 to 0.196
2	75th	0.785	0.178	0.005	0.052 to 0.303
3	25th	0.675	0.151	<0.001	0.092 to 0.209
3	50th	0.785	0.202	<0.001	0.146 to 0.257
3	75th	0.9275	0.268	<0.001	0.195 to 0.341
4	25th	0.55	0.152	<0.001	0.082 to 0.221
4	50th	0.65	0.182	<0.001	0.120 to 0.245
4	75th	0.82	0.234	<0.001	0.166 to 0.303

Supplementary Table S7. Influence-trimmed sensitivity for PEEK-minus-rigid contrasts.

Endpoint	Comparison	Adj diff	p	Global Holm p	Excluded	95% CI
canal	2P vs 2R	0.076	0.001	0.005	8	0.030 to 0.121
canal	3P vs 3R	0.137	<0.001	<0.001	8	0.093 to 0.181
canal	4P vs 4R	0.137	<0.001	0.002	8	0.062 to 0.213
disc	2P vs 2R	0.027	0.193	0.193	6	-0.014 to 0.068
disc	3P vs 3R	0.086	<0.001	<0.001	6	0.057 to 0.114
disc	4P vs 4R	0.084	<0.001	0.004	6	0.035 to 0.134
facet	2P vs 2R	-0.972	0.003	0.008	7	-1.606 to -0.339
facet	3P vs 3R	-1.173	<0.001	<0.001	7	-1.585 to -0.760
facet	4P vs 4R	-2.017	<0.001	<0.001	7	-2.606 to -1.429
foramen	2P vs 2R	0.086	0.010	0.019	8	0.021 to 0.151
foramen	3P vs 3R	0.227	<0.001	<0.001	8	0.170 to 0.285

foramen	4P vs 4R	0.189	<0.001	<0.001	8	0.124 to 0.254

Supplementary Table S8. Influence-trimmed sensitivity for preoperative degenerative-marker associations.

Endpoint	Predictor	Adj diff	p	Global Holm p	Excluded	95% CI
canal	facet effusion (yes vs no)	-0.043	0.027	0.192	7	-0.081 to -0.005
canal	Modic change (yes vs no)	0.019	0.336	1.000	7	-0.020 to 0.058
disc	facet effusion (yes vs no)	-0.040	0.001	0.009	7	-0.064 to -0.016
disc	Modic change (yes vs no)	-0.004	0.841	1.000	7	-0.042 to 0.034
facet	facet effusion (yes vs no)	-0.209	0.274	1.000	7	-0.584 to 0.166
facet	Modic change (yes vs no)	0.457	0.138	0.826	7	-0.147 to 1.061
foramen	facet effusion (yes vs no)	-0.011	0.644	1.000	8	-0.060 to 0.037
foramen	Modic change (yes vs no)	-0.025	0.308	1.000	8	-0.074 to 0.023

Supplementary Table S9. Supportive mixed-model difference-in-change contrasts for rod material comparisons.

Endpoint	Segments	Comparison	Diff in change	p	Global Holm p	95% CI
canal	2	2P vs 2R	0.070	0.047	0.049	0.001 to 0.138
canal	3	3P vs 3R	0.158	<0.001	<0.001	0.106 to 0.209
canal	4	4P vs 4R	0.156	<0.001	<0.001	0.091 to 0.220
disc	2	2P vs 2R	0.076	0.018	0.049	0.013 to 0.139
disc	3	3P vs 3R	0.091	<0.001	0.001	0.044 to 0.139
disc	4	4P vs 4R	0.084	0.006	0.023	0.024 to 0.143
facet	2	2P vs 2R	-1.689	<0.001	0.002	-2.624 to -0.754
facet	3	3P vs 3R	-1.247	<0.001	0.003	-1.952 to -0.543
facet	4	4P vs 4R	-2.045	<0.001	<0.001	-2.923 to -1.168
foramen	2	2P vs 2R	0.131	0.016	0.049	0.024 to 0.239
foramen	3	3P vs 3R	0.226	<0.001	<0.001	0.145 to 0.307
foramen	4	4P vs 4R	0.232	<0.001	<0.001	0.131 to 0.333

Supplementary Table S10. Supportive mixed-model difference-in-change contrasts for preoperative degenerative markers.

Endpoint	Predictor	Diff in change	p	Global Holm p	95% CI
canal	facet effusion (yes vs no)	-0.034	0.086	0.599	-0.073 to 0.005
canal	Modic change (yes vs no)	0.031	0.287	1.000	-0.026 to 0.087
disc	facet effusion (yes vs no)	-0.037	0.039	0.311	-0.073 to -0.002
disc	Modic change (yes vs no)	0.024	0.372	1.000	-0.028 to 0.075
facet	facet effusion (yes vs no)	-0.032	0.906	1.000	-0.562 to 0.499
facet	Modic change (yes vs no)	0.584	0.140	0.842	-0.192 to 1.359
foramen	facet effusion (yes vs no)	0.001	0.983	1.000	-0.061 to 0.062
foramen	Modic change (yes vs no)	-0.018	0.696	1.000	-0.108 to 0.072

Supplementary Table S11. Exploratory facet-effusion-by-rod-material interaction terms.

Endpoint	Interaction beta	p	Role	95% CI
canal	0.073	0.086	not for primary inference	-0.010 to 0.155
disc	0.059	0.035	not for primary inference	0.004 to 0.114
facet	-0.249	0.631	not for primary inference	-1.263 to 0.765

foramen	0.002	0.962	not for primary inference	-0.098 to 0.103

Supplementary Table S12. Composite any-preoperative-degenerative-finding sensitivity analysis.

Endpoint	Adj diff	p	Global Holm p	95% CI
foramen	-0.005	0.867	1.000	-0.058 to 0.049
canal	-0.019	0.358	1.000	-0.061 to 0.022
disc	-0.027	0.074	0.295	-0.056 to 0.003
facet	0.097	0.708	1.000	-0.412 to 0.607

Supplementary Table S13. Descriptive within-group paired pre-to-postoperative tests.

Group	Endpoint	n	Pre mean (SD)	Post mean (SD)	Delta mean (SD)	Paired t p	Wilcoxon p
2P	canal	11	1.025 (0.180)	1.028 (0.173)	0.004 (0.032)	0.712	0.930
2R	canal	16	1.107 (0.188)	1.057 (0.182)	-0.050 (0.055)	0.003	0.008
3P	canal	18	1.203 (0.156)	1.212 (0.148)	0.008 (0.076)	0.650	0.981
3R	canal	32	1.294 (0.288)	1.135 (0.275)	-0.159 (0.094)	<0.001	<0.001
4P	canal	12	1.100 (0.118)	1.068 (0.118)	-0.032 (0.039)	0.015	0.028
4R	canal	17	1.299 (0.238)	1.117 (0.301)	-0.182 (0.136)	<0.001	<0.001
2P	disc	11	0.820 (0.316)	0.817 (0.309)	-0.003 (0.040)	0.825	0.645
2R	disc	16	0.964 (0.190)	0.894 (0.225)	-0.070 (0.104)	0.017	0.012
3P	disc	18	0.947 (0.155)	0.946 (0.144)	-0.001 (0.030)	0.877	0.102
3R	disc	32	0.958 (0.244)	0.858 (0.207)	-0.100 (0.095)	<0.001	<0.001
4P	disc	12	0.886 (0.218)	0.870 (0.219)	-0.016 (0.025)	0.054	0.066
4R	disc	17	0.822 (0.215)	0.725 (0.221)	-0.098 (0.093)	<0.001	0.002
2P	facet	11	6.333 (1.586)	6.224 (1.330)	-0.109 (0.595)	0.557	0.832
2R	facet	16	6.188 (2.091)	7.575 (2.144)	1.387 (1.915)	0.011	0.009
3P	facet	18	5.725 (1.493)	5.743 (1.415)	0.018 (0.225)	0.741	0.068
3R	facet	32	5.193 (1.491)	6.637 (1.691)	1.444 (1.375)	<0.001	<0.001
4P	facet	12	5.192 (1.790)	5.255 (1.745)	0.063 (0.288)	0.462	0.032
4R	facet	17	5.298 (1.531)	7.379 (1.633)	2.082 (1.093)	<0.001	<0.001
2P	foramen	11	0.565 (0.142)	0.576 (0.136)	0.011 (0.043)	0.420	0.656
2R	foramen	16	0.682 (0.219)	0.564 (0.160)	-0.119 (0.185)	0.021	0.031
3P	foramen	18	0.867 (0.223)	0.849 (0.227)	-0.018 (0.036)	0.050	0.029
3R	foramen	32	0.790 (0.226)	0.543 (0.194)	-0.247 (0.166)	<0.001	<0.001
4P	foramen	12	0.632 (0.158)	0.607 (0.154)	-0.025 (0.038)	0.046	0.068
4R	foramen	17	0.782 (0.292)	0.524 (0.235)	-0.258 (0.147)	<0.001	<0.001

These tests are descriptive within-group summaries and are not between-group PEEK-versus-rigid evidence.

Supplementary Table S14. Reproducibility and output validation checks.

Check	Status
Data-sheet equivalence, rod/construct notebook	True
Data-sheet equivalence, degenerative-marker notebook	True
Expected outputs valid, rod/construct notebook	True
Expected outputs valid, degenerative-marker notebook	True
Table-shell compliance, rod/construct table	True
Table-shell compliance, degenerative-marker table	True

Supplementary Figure S1. Crude preoperative-to-postoperative mean profiles by construct group.

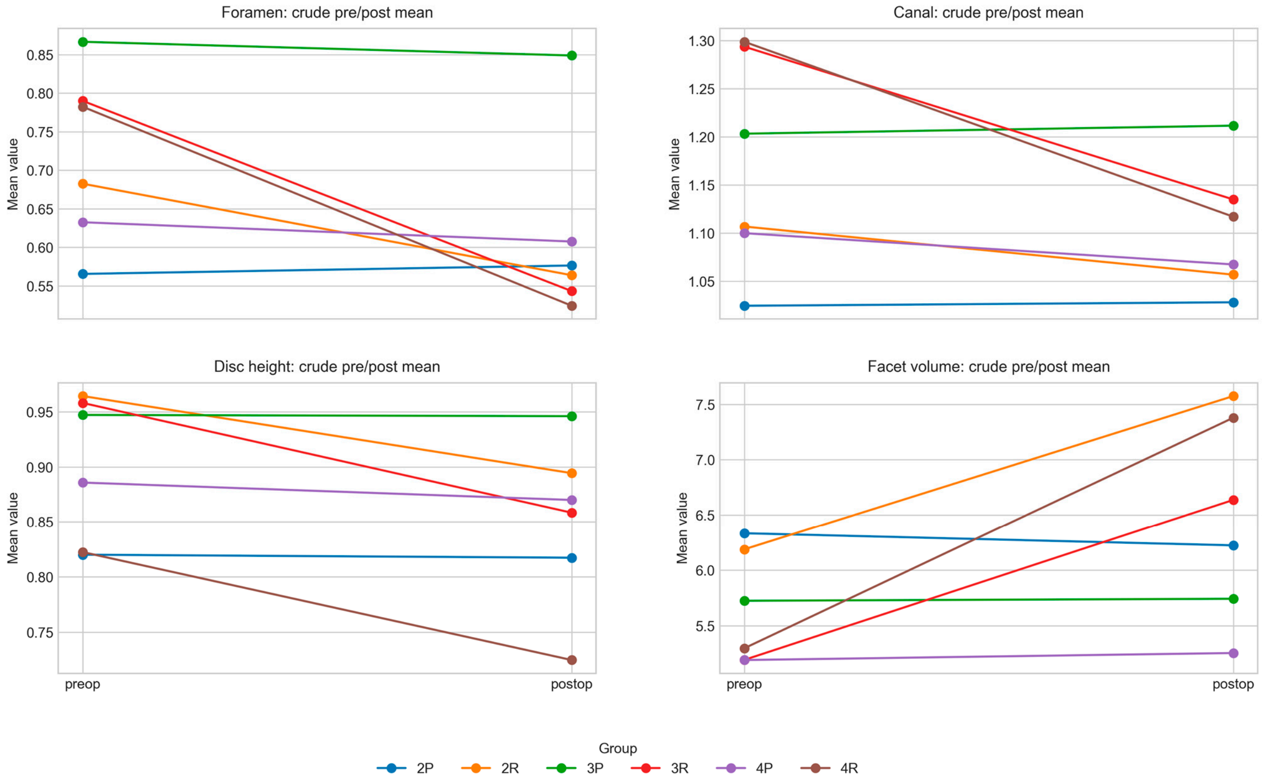

Supplementary Figure S2. Change-score distributions by construct group.

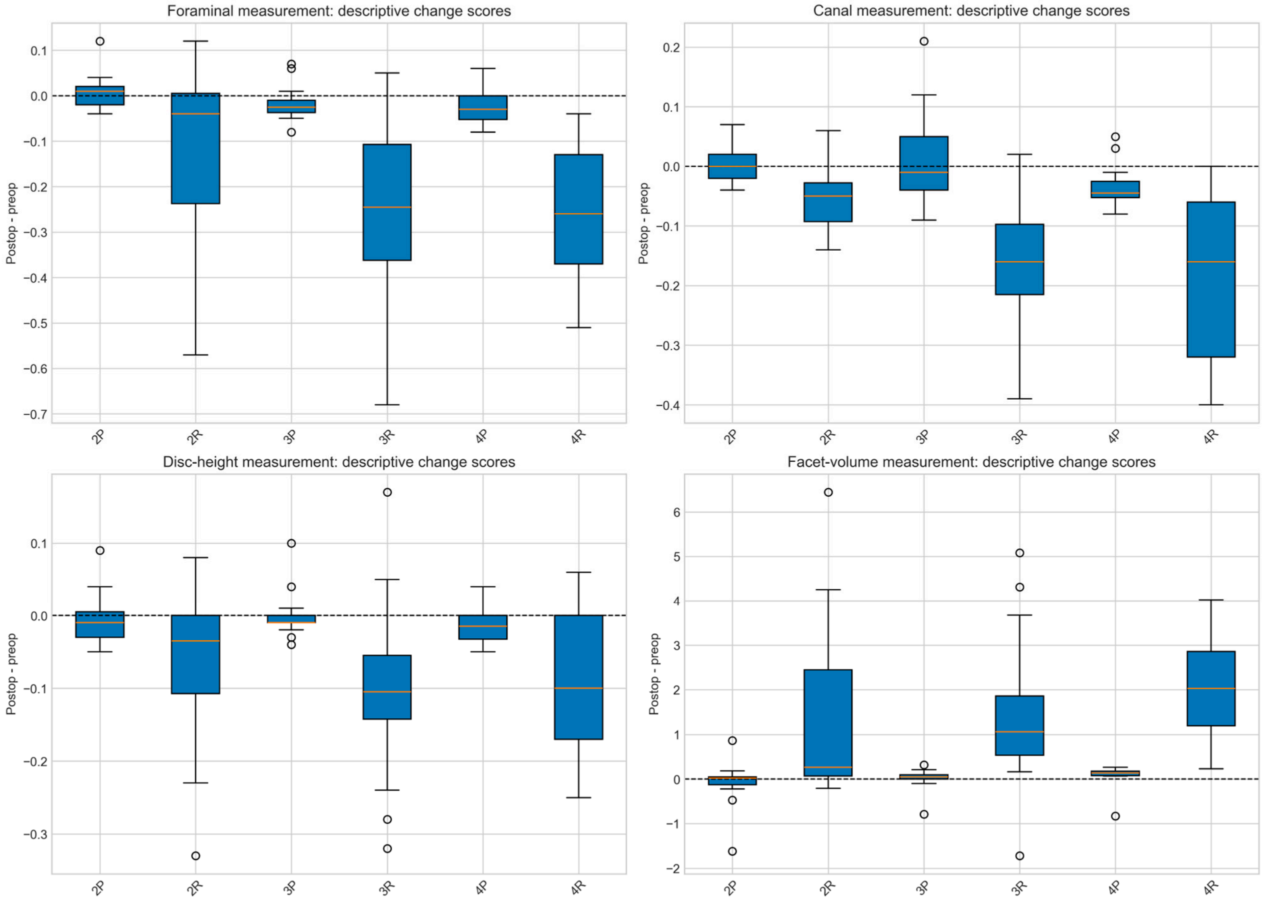

Supplementary Figure S3. Adjusted postoperative marginal means by construct group.

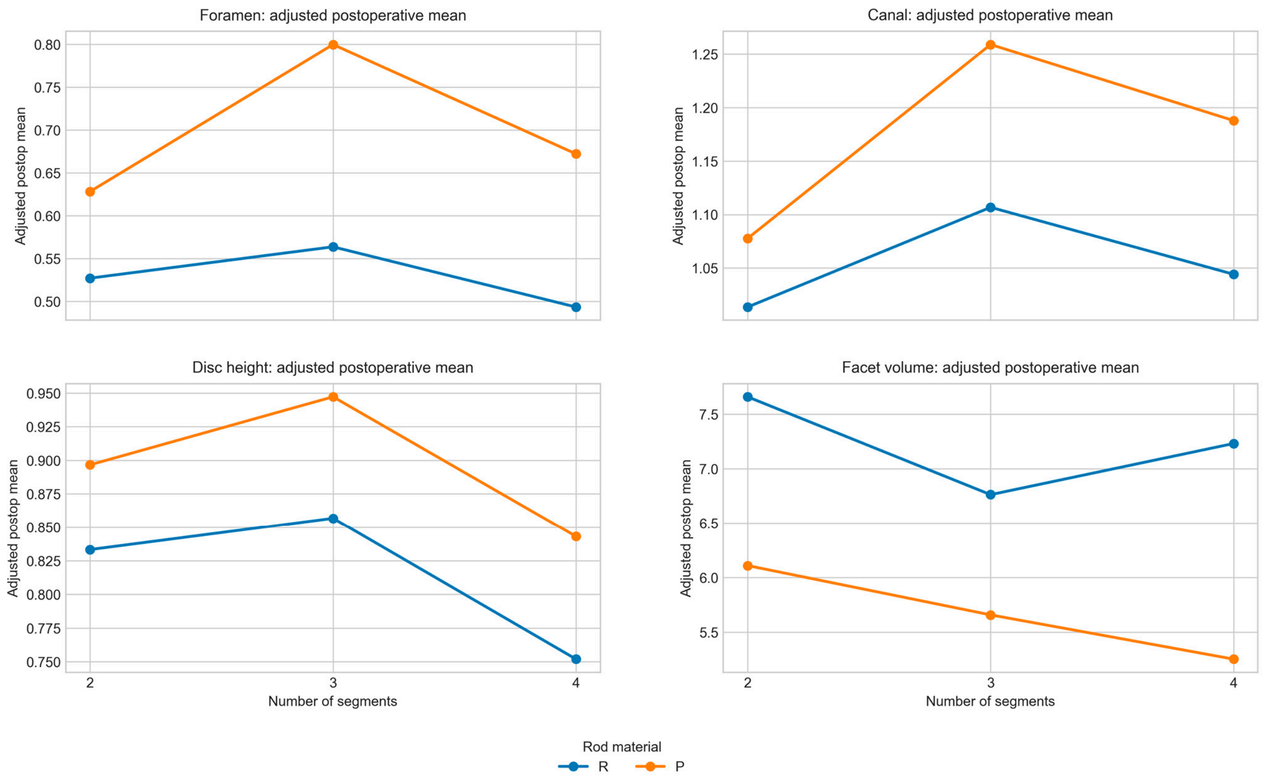

Supplementary Figure S4. Baseline-dependent foramen sensitivity estimates.

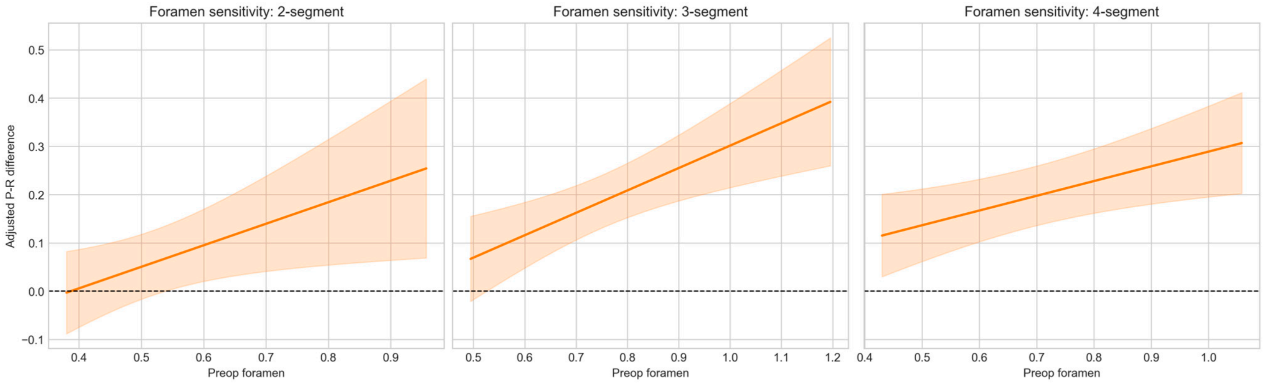

Supplementary Figure S5. Supportive mixed-model PEEK-minus-rigid difference-in-change contrasts.

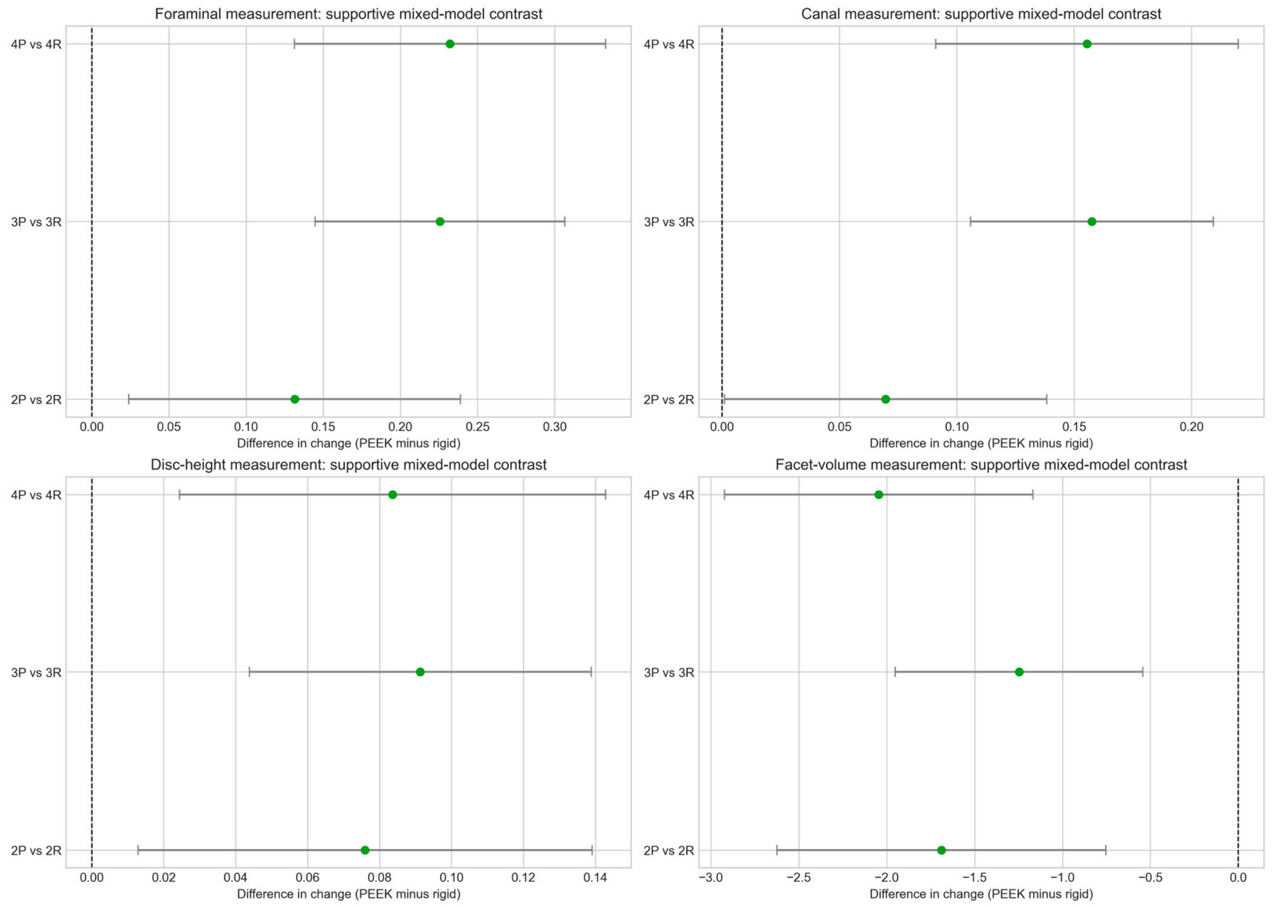

Supplementary Figure S6. Preoperative degenerative-marker prevalence by construct group.

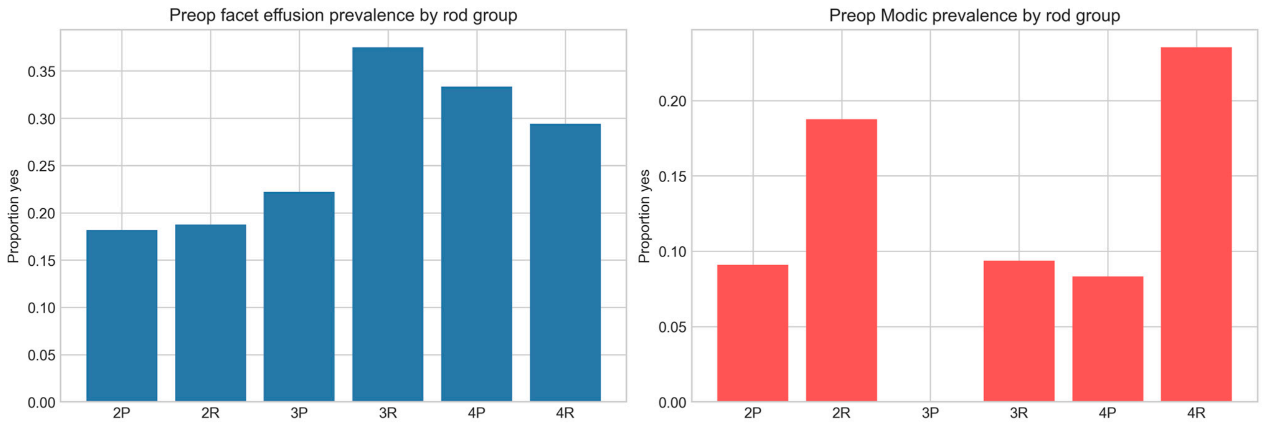

Supplementary Figure S7. Overlap of preoperative facet effusion and Modic change.

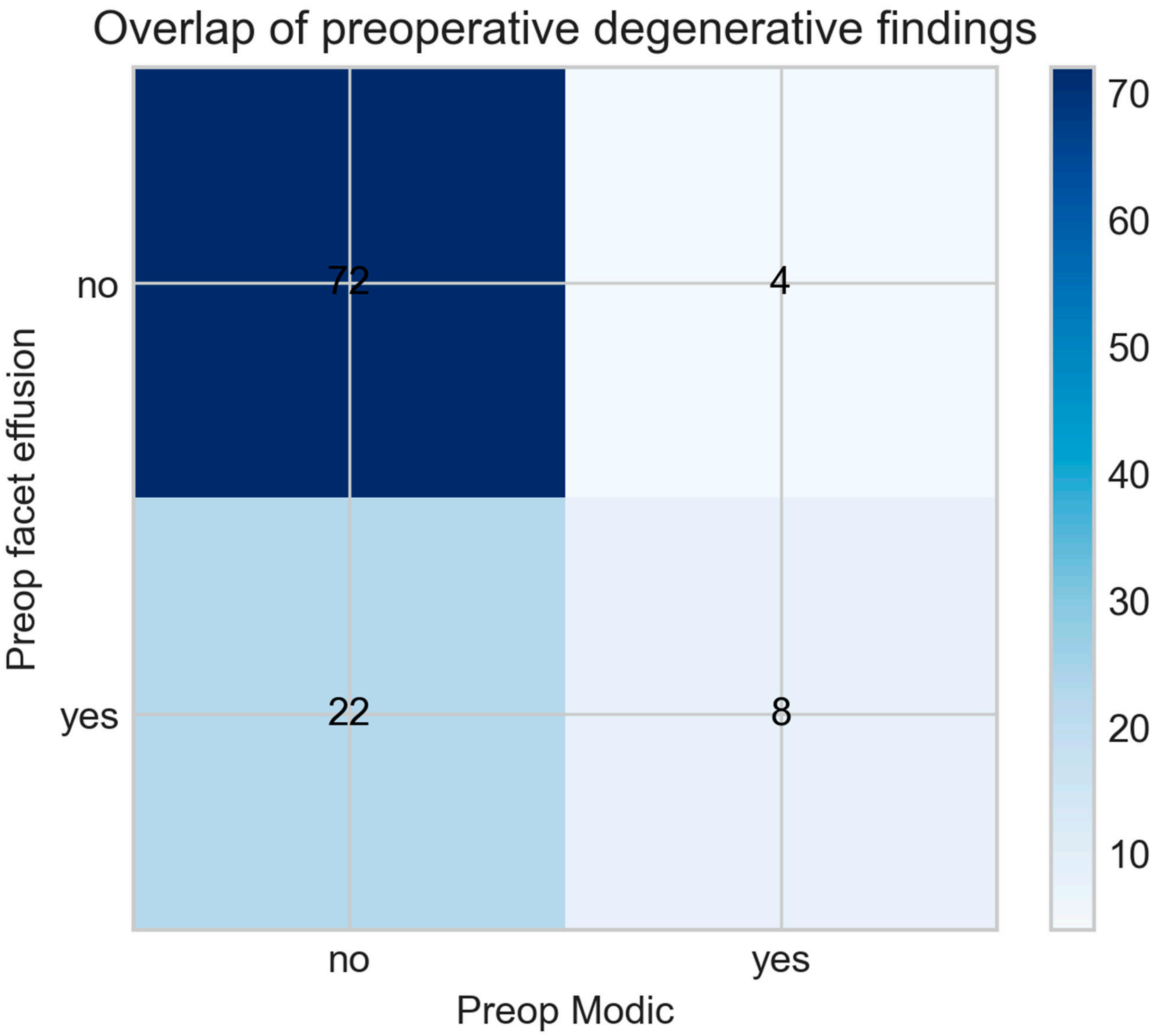

Supplementary Figure S8. Exploratory adjusted margins for facet effusion by rod material.

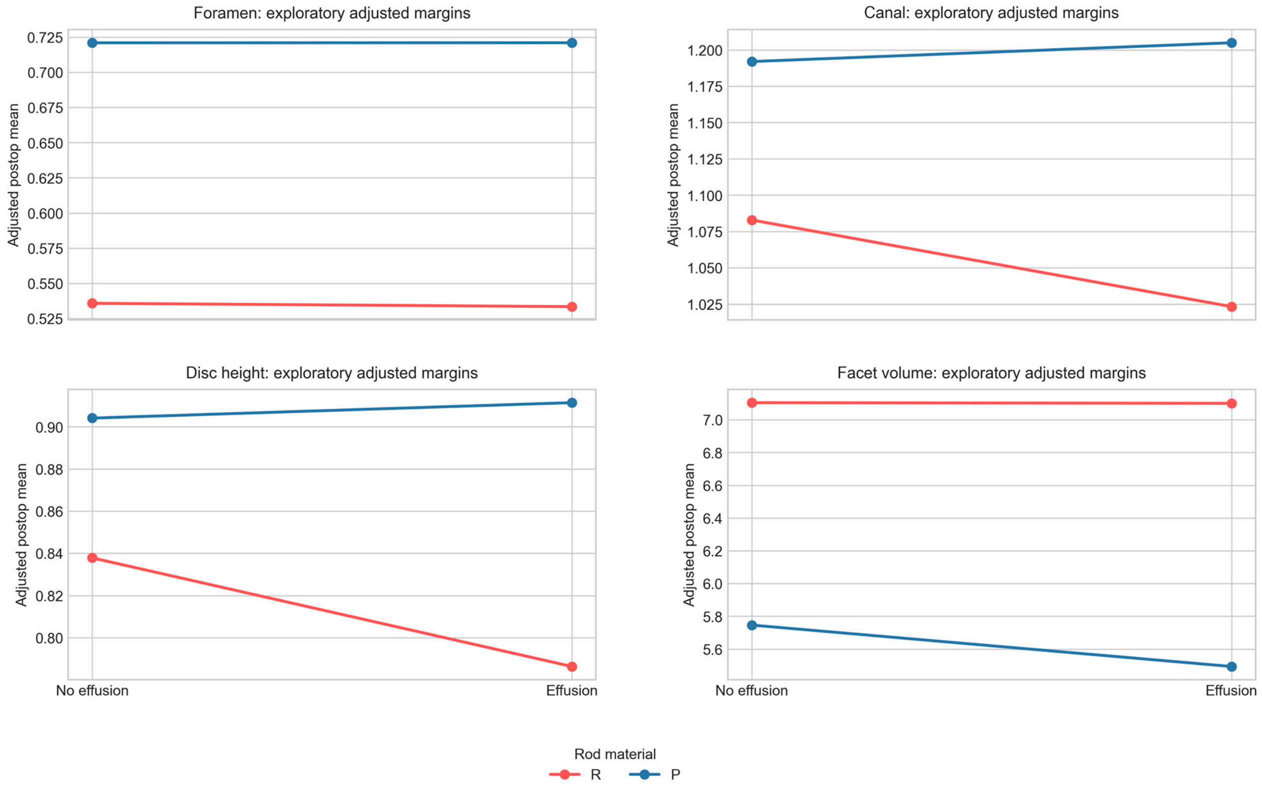

Supplementary Figure S9. Foramen exposure effect sensitivity comparison.

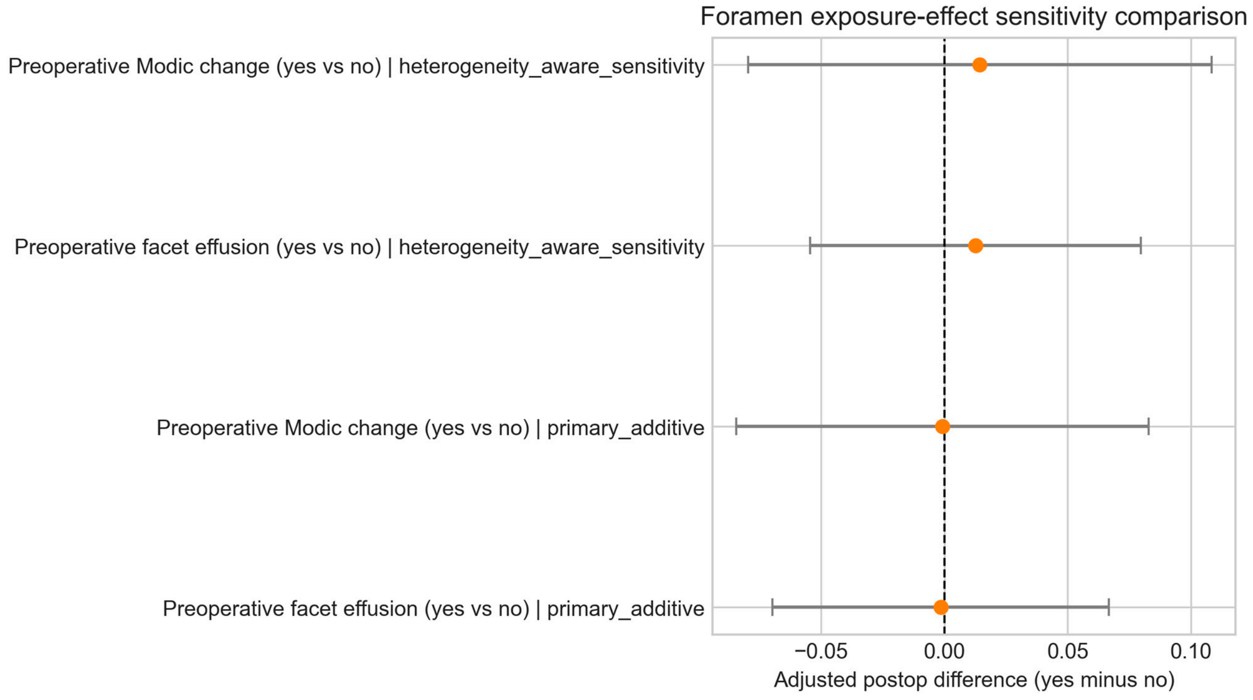

Supplementary Figure S10. Diagnostic plots for the foramen rod material model.

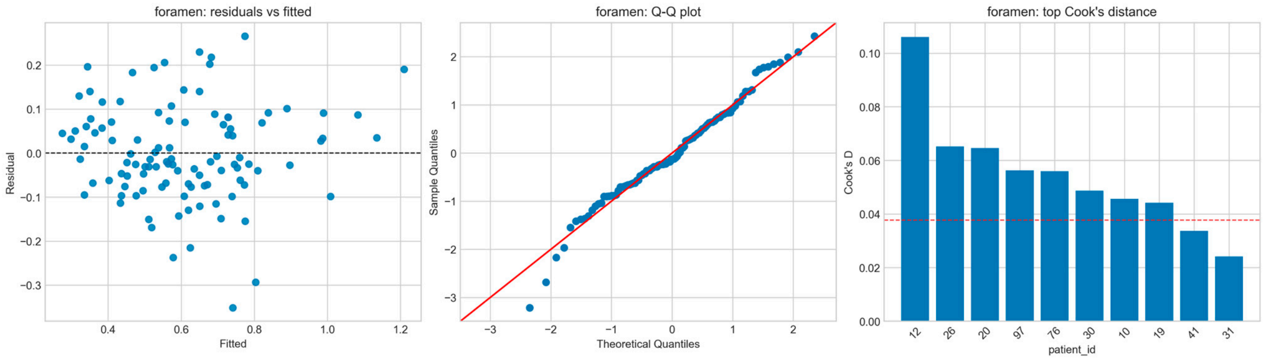

Supplementary Figure S11. Diagnostic plots for the canal rod material model.

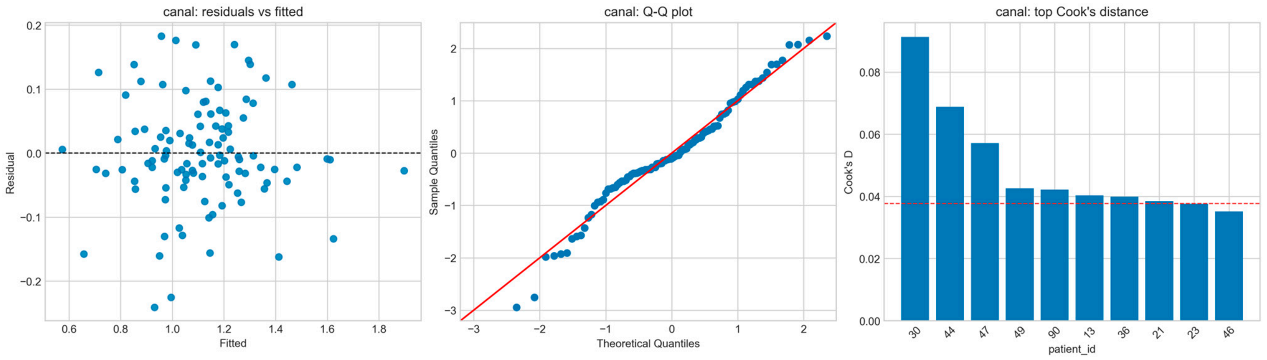

Supplementary Figure S12. Diagnostic plots for the disc rod material model.

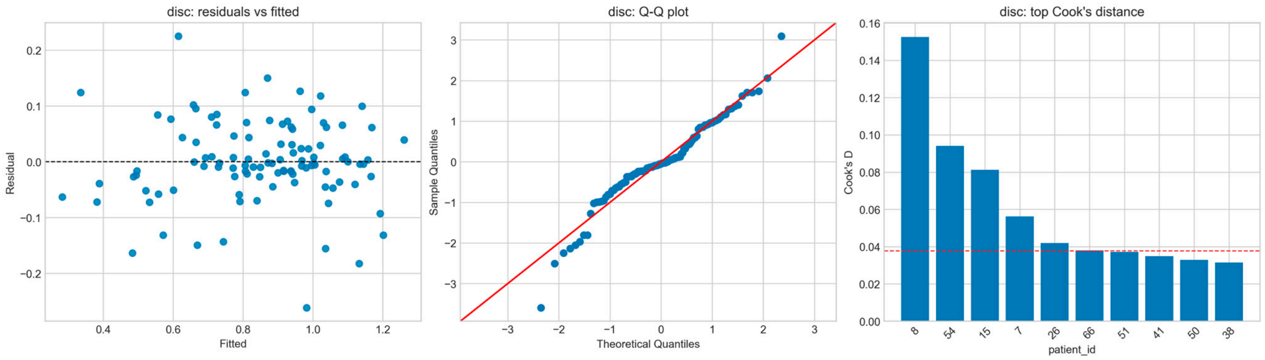

Supplementary Figure S13. Diagnostic plots for the facet rod material model.

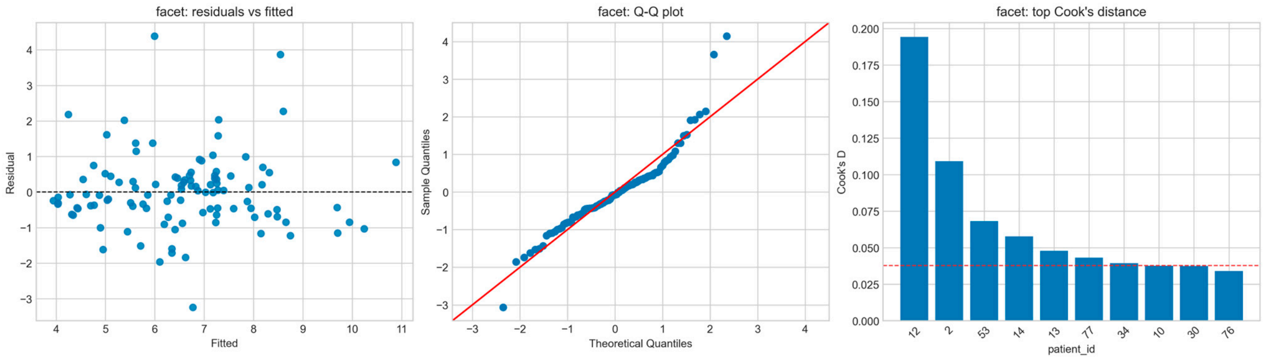

Supplementary Figure S14. Diagnostic plots for the foramen degenerative marker model.

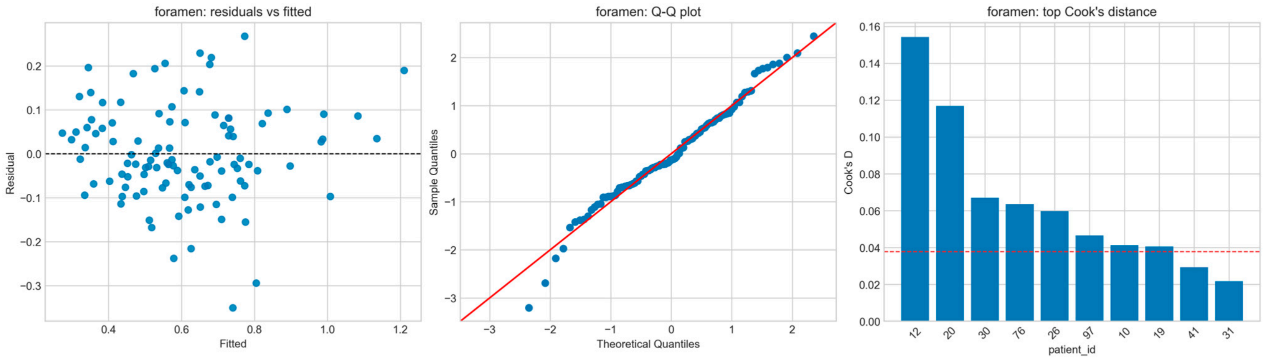

Supplementary Figure S15. Diagnostic plots for the canal degenerative marker model.

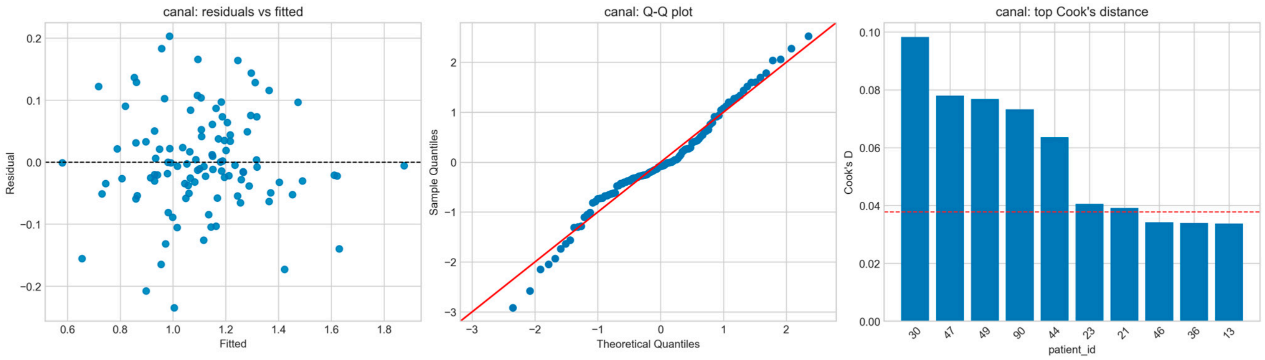

Supplementary Figure S16. Diagnostic plots for the disc degenerative marker model.

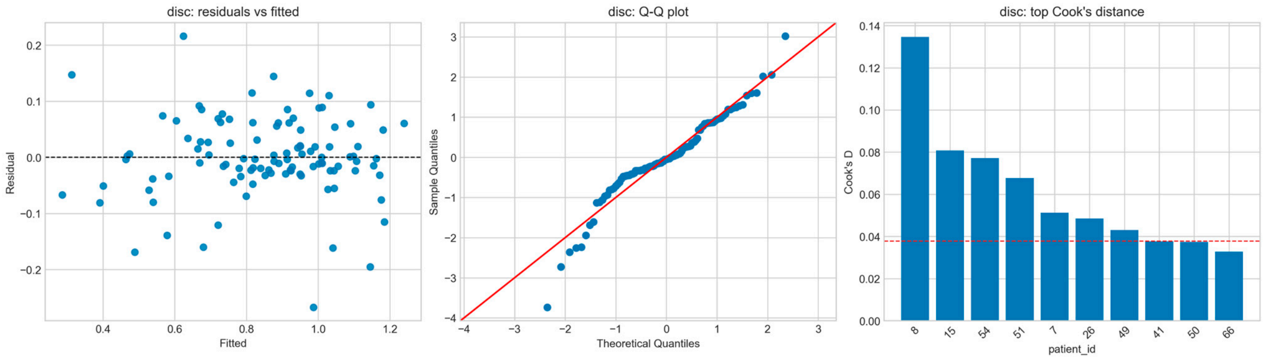

Supplementary Figure S17. Diagnostic plots for the facet degenerative marker model.

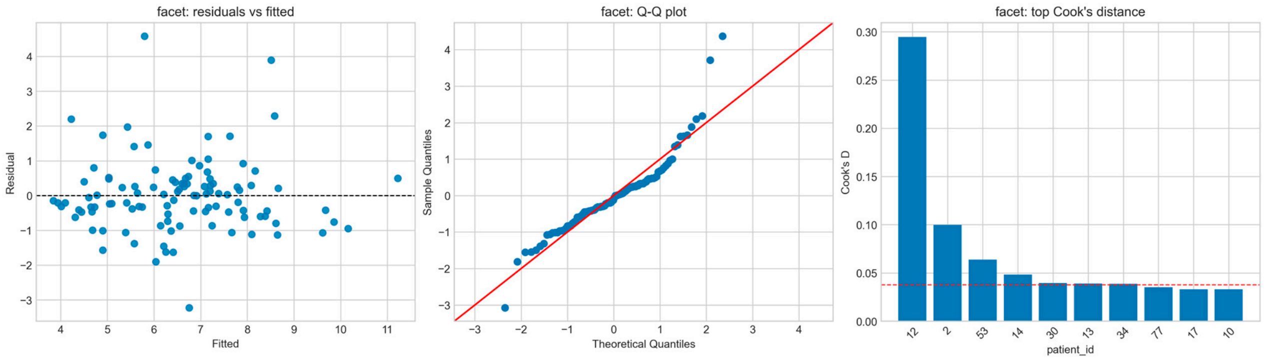

Supplement: Supplementary file 1 [file diagnostics-16-01625-s001.zip › diagnostics-4322672-supplementary.pdf]
